# Systematic competition between strain and electric field stimuli in tuning EELS of phosphorene

**DOI:** 10.1038/s41598-021-83213-0

**Published:** 2021-02-12

**Authors:** Mohsen Yarmohammadi, Bui Dinh Hoi, Le Thi Thu Phuong

**Affiliations:** 1 Department of Energy Engineering and Physics, Amirkabir University of Technology, Tehran, 14588 Iran; 2 Department of Physics, University of Education, Hue University, Hue City, Viet Nam; 3 Center for Theoretical and Computational Physics, University of Education, Hue University, Hue City, Viet Nam

**Keywords:** Two-dimensional materials, Electronic properties and materials, Phase transitions and critical phenomena, Electronic structure, Electronic devices, Nanophotonics and plasmonics

## Abstract

The strongly anisotropic properties of phosphorene makes it an attractive material for applications in deciding the specific direction for different purposes. Here we have particularly reported the competition between strain and electric field stimuli in evaluating the band gap and electron energy loss spectrum (EELS) of single-layer black phosphorus using the tight-binding method and the Kubo conductivity. We construct possible configurations for this competition and evaluate the *interband optical excitations* considering the corresponding band gap variations. The band gap increases with the individual electric field, while it increases (decreases) with tensile (compressive) uniaxial in-plane strain. Contrary to the in-plane strains, the uniaxial out-of-plane strain shows a critical strain at which the system suffers from a phase transition. Furthermore, the presence of these stimuli simultaneously results in an extraordinary band gap engineering. Based on the EELS response in the electromagnetic spectrum, the armchair (zigzag) direction is classified into the infrared and visible (ultraviolet) region. We report that the electric field gives rise to the blue shift in the interband optical transitions along the armchair direction, while the compressive/tensile (tensile/compressive) in-plane/out-of-plane strain provides a red (blue) shift. Moreover, we observe an inverse behavior of EELS response to the individual and combined effects of electric field and strains compared to the band gap behavior except at critical out-of-plane strain for which the physical theory of interband excitation is simply violated. Our results provide a new perspective on the applicability of phosphorene in stimulated optical applications.

## Introduction

After the isolation of single-layer graphene^1–4^, different kinds of two-dimensional (2D) materials with distinct functionality were discovered and, in turn, many pieces of research on the few-layer form of known bulk materials have started to develop^5–9^. One of these 2D materials is monolayer black phosphorus, obtained in 1914 in the form of bulk, and in 2014 in the form of few-layer, so-called phosphorene^10–22^. Among different allotropes of phosphorene, black phosphorus (BP) is the most stable one, which possesses a nonplanar/puckered honeycomb lattice unlike planar graphene, transition-metal dichalcogenides and hexagonal boron nitride^23^. Additionally, BP presents a highly tunable anisotropic dispersion of fermions with a direct band gap of about 1.5–2 eV located at the $\Gamma$ point of the first Brillouin zone (FBZ)^23–25^. The direct band gap in BP can be tuned through the number of layers and doping^15–17^. Also, researchers have reported a high on-off current ratio and high mobility for charge carriers in field-effect transistors based on BP^10,21,26^. All these together lead to tremendous research activities on the electronic and optical properties of BP^27–29^.

The excellent response of 2D crystals to the external mechanical and electrical stimuli stems from their fantastic tunable optoelectronics^30,31^. Meanwhile, tuning BP features in optoelectronics has been extensively studied by strain^32^, electric field^33–37^ and other several factors^15,24,38–40^. The interband optical transitions of few-layer BP have been experimentally studied by Li et al.^41^ to cover a wide technologically important spectral range from the visible to the mid-infrared region. On the other hand, the photoluminescence^10,42^ (an extraordinary photoluminescence peak at 1.45 eV), Raman spectra^26^, photocurrent generation^43^, and optical absorption^44^ of BP have been measured experimentally. The highly anisotropic nature of phosphorene has been demonstrated through Raman and polarization photoluminescence measurements, in which the photoluminescence spectroscopy has also revealed the layer-dependent band gap of phosphorene^18^. Theoretical point of view, it has been shown that the uniaxial strain along both armchair and zigzag directions of few-layer BP modulates significantly the linear dichroism and the Faraday rotation quantities^45^. Furthermore, the optical conductivity of BP in the presence of both uni- and bi-axial strains has been theoretically calculated using the Kubo formula^46^. In addition to the external mechanical effects, it has been reported that the linear dichroism and optical conductivity of few-layer BP can be controlled by the perpendicular electric field^47^. In another work, tuning optical properties of phosphorene by adsorption of alkali metals (Li and Na) and halogens (Br and Cl) has been investigated^48^. The authors have found that increasing the size of alkali metals and halogen adsorbed onto the phosphorene layer leads to the reduction of the absorption coefficient and the shift of the absorption peak towards the visible region. Also, employing first-principles calculations based on density functional theory (DFT), the electronic and optical properties of phosphorene co-doped with vanadium and nonmetallic atoms (B, C, N, and O) have been investigated^49^. In these doped systems, an interesting red shift phenomenon can be observed. In other works, perpendicular electric field effects on the propagation of electromagnetic waves through the monolayer phosphorene^50^, optical interband transitions, and optical activity in strained phosphorene^51^ and blue shift in the interband optical transitions of gated monolayer black phosphorus^52^ have been addressed well.

Accordingly, there are various methods to tune the optical properties of phosphorene. Furthermore, for broader optoelectronic applications further details of the optical properties of phosphorene are still in demand. The electron energy loss spectrum (EELS) is one of the ideal tools to study electronic excitations in materials for transmission electron microscopy measurements^53–55^. This quantity tells us how the energy of scattered host electrons is changed from the external perturbations, in which this, in turn, characterizes the inelastic scattering process. A systematic study of EELS of BP in the presence of both electrical and mechanical stimuli is still missing in the literature, knowledge that is still incomplete up until now. Thus, in the present work, a central question in the field of BP optoelectronics research is how the competition between the external electrical and mechanical stimuli affects the EELS of BP.

Our approach follows a simple, but effective, two-band tight-binding Hamiltonian^19,46,56,57^ in the presence of both strain and electric field to explain the electronic and optical properties of BP considering the atomic units. We use the Kubo formula within the linear response theory to calculate both real and imaginary parts of the interband optical conductivity of BP in full agreement with other works. However, we finally calculate the dielectric function and EELS systematically. As we will see, the numerical calculations propose critical strains and electric fields for which the EELS is maximized and minimized, which are extremely useful in real applications.

This paper is organized as follows: in the next section we present the tight-binding Hamiltonian model of pristine BP. Then, we introduce a simple model for strain and electric field-induced BP; perturbed Hamiltonian. In “Interband optical conductivity, dielectric function and EELS of phosphorene”, we present the necessary formalism to compute the interband optical conductivity employing the Kubo formula. Next, we introduce the dielectric function and corresponding EELS. Afterward, the systematical numerical results are given by “Results and discussions”. “Conclusions” concludes the paper.

## Tight-binding Hamiltonian model

### Four-band to two-band model of pristine phosphorene

**Figure 1:**
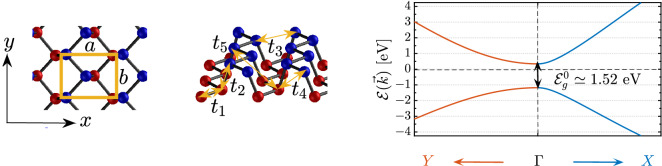
Left side, top, and full view of single-layer phosphorene, respectively. The unit cell of phosphorene is shown with an orange rectangle with *a* and *b* as the length of the unit cell along the *x* and *y* direction, respectively. On the right side, the band structure of the two-band model of pristine phosphorene is represented with the band gap of $\mathcal {E}^0_g$ located at the $\Gamma$ point of the FBZ.

The band structure of phosphorene can be explored as other 2D materials as a continuous deformation of the honeycomb lattice by different hopping energies $t_i$ over the five neighbors, as shown on the left side of Fig. 1. As can be seen from the top view geometry, the unit cell of monolayer BP contains two sublayers with four atoms, two in the lower sublayer, and two in the upper sublayer (see the full view). The effective tight-binding Hamiltonian of BP in the absence of strain and the electric field is introduced based on $G_0 W_0$ approximation as^19,56,57^:

1 $$\begin{aligned} \mathcal {H}=\sum _{i, s} t^0_{i,s} \hat{f}^{\dagger }_{i,s}\hat{f}_{i,s} + \sum _{\langle i,j \rangle , s} t_{ij}\hat{f}^{\dagger }_{i,s}\hat{f}_{j,s}+\text {H.c.}\,, \end{aligned}$$

where the summation runs over the five nearest neighbor lattice sites. Also, $t^0_{i,s}$ is the on-site energy of electron at site *i* of sublayer *s* and $t_{ij}$ refers to the intrasublayer and intersublayer hoping energy of electron from site *i* to the site *j*. On the other hand, $\hat{f}^{\dagger }_{i,s}$ and $\hat{f}_{i,s}$ is the creation and annihilation operator at site *i* in the sublayer *s*, while H.c. stands for the Hermitian conjugate of the second term. From the $G_0 W_0$ approximation performed in Ref.^56^, one finds $t_{1}=-1.220$ eV, $t_{2}=+3.665$ eV, $t_{3}=-0.205$ eV, $t_{4}=-0.105$ eV, and $t_{5}=-0.055$ eV. We set $t^0_i$ to zero throughout the manuscript for all *sp* states. Let us define the field operator ${\Psi}^{\dagger}_{\mathbf {k}} = \Big[ a^{\dagger}_{\mathbf {k}},b^{\dagger}_{\mathbf {k}},c^{\dagger}_{\mathbf {k}},d^{\dagger}_{\mathbf {k}}\Big] ^{\mathrm{T}}$ to obtain the momentum representation of the Hamiltonian in Eq. (1) as $\mathcal {H}=\sum _{\mathbf {k}}\psi ^{\dagger }_{\mathbf {k}}\mathcal {H}(\mathbf {k})\psi _{\mathbf {k}}$ in which

2 $${\mathcal{H}}({\mathbf{k}}) = \left( {\begin{array}{*{20}l} 0 \hfill & {t_{2} f_{2} ({\mathbf{k}}) + t_{5} f_{5} ({\mathbf{k}})} \hfill & {t_{4} f_{4} ({\mathbf{k}})} \hfill & {t_{1} f_{1} ({\mathbf{k}}) + t_{3} f_{3} ({\mathbf{k}})} \hfill \\ {t_{2} f_{2}^{*} ({\mathbf{k}}) + t_{5} f_{5}^{*} ({\mathbf{k}})} \hfill & {0} \hfill & {t_{1} f_{1}^{*} ({\mathbf{k}}) + t_{3} f_{3}^{*} ({\mathbf{k}})} \hfill & {t_{4} f_{4}^{*} ({\mathbf{k}})} \hfill \\ {t_{4} f_{4}^{*} ({\mathbf{k}})} \hfill & {t_{1} f_{1} ({\mathbf{k}}) + t_{3} f_{3} ({\mathbf{k}})} \hfill & {0} \hfill & {t_{2} f_{2} ({\mathbf{k}}) + t_{5} f_{5} ({\mathbf{k}})} \hfill \\ {t_{1} f_{1}^{*} ({\mathbf{k}}) + t_{3} f_{3}^{*} ({\mathbf{k}})} \hfill & {t_{4} f_{4} ({\mathbf{k}})} \hfill & {t_{2} f_{2}^{*} ({\mathbf{k}}) + t_{5} f_{5}^{*} ({\mathbf{k}})} \hfill & {0} \hfill \\ \end{array} } \right),$$

where the structure factors are given by

3a $$\begin{aligned} f_1(\mathbf {k}) =&2 e^{\mathtt{i} k_x a/2\sqrt{3}} \cos \left[ k_y b/2\right] ,\quad f_2(\mathbf {k}) = {} e^{- \mathtt{i} k_x a/\sqrt{3}},\quad f_3(\mathbf {k}) = 2 e^{-\mathtt{i} 5k_x a/2\sqrt{3}} \cos \left[ k_y b/2\right] , \end{aligned}$$

3b $$\begin{aligned} f_4(\mathbf {k}) =&4 \cos \left[ \sqrt{3} k_x a/2\right] \cos \left[ k_y b/2\right] ,\quad f_5(\mathbf {k}) = e^{2 \mathtt{i} k_x a/\sqrt{3}}. \end{aligned}$$

with $a=4.43$ Å  the length of the unit cell along the armchair direction and $b=3.27$ Å  the length of the unit cell along the zigzag direction^46,56,57^.

It is well-known that the phosphorene structure possesses a $D_{2h}$ point group symmetry^19,56^ and allows us to reduce the dimension of the Hamiltonian from four to two such that the principle physics of the system is preserved well and the analytical calculations are simplified. This can be understood conceptually from the geometry structure of phosphorene. If one sits on one of the phosphorus atoms in the upper sublayer, no difference is felt when sitting on one of the atoms from the lower sublayer. So, we reduce the unit cell to two atoms and write the two-band Hamiltonian model as^19,56^

4 $${\mathcal{H}}({\mathbf{k}}) = \left( {\begin{array}{*{20}l} {t_{4} f_{4} ({\mathbf{k}})} \hfill & {t_{1} f_{1} ({\mathbf{k}}) + t_{2} f_{2} ({\mathbf{k}}) + t_{3} f_{3} ({\mathbf{k}}) + t_{5} f_{5} ({\mathbf{k}})} \hfill \\ {t_{1} f_{1}^{*} ({\mathbf{k}}) + t_{2} f_{2}^{*} ({\mathbf{k}}) + t_{3} f_{3}^{*} ({\mathbf{k}}) + t_{5} f_{5}^{*} ({\mathbf{k}})} \hfill & {t_{4} f_{4} ({\mathbf{k}})} \hfill \\ \end{array} } \right),$$

By this, we find the energy dispersion as

5 $$\begin{aligned} \mathcal {E}_{\pm }(\mathbf {k}) = t_4 f_4(\mathbf {k}) \pm \sqrt{g(\mathbf {k})g^*(\mathbf {k})}, \end{aligned}$$

where $g(\mathbf {k}) = t_1 f_1(\mathbf {k}) + t_2 f_2(\mathbf {k}) + t_3 f_3(\mathbf {k}) + t_5 f_5(\mathbf {k})$ and $+(-)$ refers to the conduction (valence) band. To have the electronic phase of the system, we need the band gap. As explained before, the band gap is located at $\Gamma$ point of the FBZ, i.e. at $k_x = k_y = 0$, so the pristine band gap is obtained as

6 $$\begin{aligned} \mathcal {E}^0_g = 2\left( 2t_1 + t_2 + 2t_3 + t_5\right) = 1.52\, \mathrm{eV}. \end{aligned}$$

Obviously the FBZ of phosphorene is also a rectangle with coordinates $k_x \in [-2\pi /a,2\pi /a]$ and $k_y \in [-2\pi /b,2\pi /b]$. In the center of FBZ, we have the $\Gamma$ point, while *X* (*Y*) point reside along the *x* (*y*) direction. Figure 1, right panel, shows the electronic band structure along the high symmetry points $\Gamma -X$ and $\Gamma -Y$ of the FBZ of pristine phosphorene using Eq. (5). Highly anisotropic structure stemming from the anisotropic carrier Fermi velocities and effective masses along the different directions can be observed, in good agreement with Refs.^19,46,56,57^ with the band gap of about $\mathcal {E}^0_g=$1.52 eV. This band anisotropy leads to the direction-dependent interband optical transitions^46^ and eventually different dielectric function and EELS.

### Strained and gated two-band model of phosphorene

In this section, we intend to describe the Hamiltonian of monolayer BP in the presence of both strain and gate voltage:

7 $$\begin{aligned} \mathcal {H}= \sum _{\langle i,j \rangle , s} \tilde{t\,}_{ij}\hat{f}^{\dagger }_{i,s}\hat{f}_{j,s}+\frac{1}{2}\sum _{i, s}\mathcal {G}_{s}\hat{f}^{\dagger }_{i,s}\hat{f}_{i,s}+\text {H.c.} = \sum _{\langle i,j \rangle , s}\underbrace{t_{ij}\exp \left[ \frac{\mathtt{i}\, e\, x_{ij}A_x(t_{ij})}{\hbar }\right] }_{:=\tilde{t}_{ij}}\hat{f}^{\dagger }_{i,s}\hat{f}_{j,s}+\frac{1}{2}\sum _{i, s}\mathcal {G}_{s}\hat{f}^{\dagger }_{i,s}\hat{f}_{i,s} +\text {H.c.}, \end{aligned}$$

where $\tilde{t}_{ij}$ is used for the strain-induced hopping, *e* is the electron charge, $x_{ij}$ is the distance between *i* and *j* sites along the *x*-direction [after applying strain] and $A_x(t_{ij})$ is the vector potential induced by the pseudomagnetic field originated from the strain. Also, $\mathcal {G}_{s}$ refers to the applied gate voltage on the top and bottom of sublayer *s* with $+V$ and $-V$ potentials, respectively. Interestingly, a single top (bottom) gate is applied to phosphorene by self-consistent tight-binding calculations, leading to the *n*-type (*p*-type) doped system with a finite density of electrons (holes) in the conduction (valence) band^58^. In this case, the charge screening effects due to the gating becomes important for the intraband optical transitions and can be neglected for the interband ones. Hence, the matrix Hamiltonian of the strained and gated BP can be written as

8 $${\mathcal{H}}({\mathbf{k}}) = \left( {\begin{array}{*{20}l} {\tilde{t}_{4} f_{4} ({\mathbf{k}}) + V/2} \hfill & {\tilde{t}_{1} f_{1} ({\mathbf{k}}) + \tilde{t}_{2} f_{2} ({\mathbf{k}}) + \tilde{t}_{3} f_{3} ({\mathbf{k}}) + \tilde{t}_{5} f_{5} ({\mathbf{k}})} \hfill \\ {\tilde{t}_{1} f_{1}^{*} ({\mathbf{k}}) + \tilde{t}_{2} f_{2}^{*} ({\mathbf{k}}) + \tilde{t}_{3} f_{3}^{*} ({\mathbf{k}}) + \tilde{t}_{5} f_{5}^{*} ({\mathbf{k}})} \hfill & {\tilde{t}_{4} f_{4} ({\mathbf{k}}) - V/2} \hfill \\ \end{array} } \right),$$

As for the strained hoppings, we mention that the variation of the bond lengths and bond angles in the presence of strain leads to the modulation of the hopping energies^59,60^. Simply, the initial coordinates of atomic sites *i*, i.e. $\mathbf {r}_{i\,\beta }$ for $\beta =\{x,y,z\}$ can be transferred to the coordinates $\tilde{\mathbf {r}}_{i\,\beta }$ through

9 $$\begin{aligned} \tilde{\mathbf {r}}_{i\,\beta }=\left[ 1+\epsilon _{\beta }\right] \mathbf {r}_{i\,\beta }, \end{aligned}$$

where $\epsilon _{\beta }$ is the strain modulus along the $\beta$-direction. Finally, we achieve the following relation for the norm of $\tilde{r}_{i}$ in the linear deformation regime

10 $$\begin{aligned} \tilde{r}_{i}=\Big[ 1+\sum _{\beta }\alpha _{i}^{\beta }\varepsilon _{\beta }\Big] r_{i}, \end{aligned}$$

where dimensionless geometrical coefficients $\alpha _{i}^{\beta }=(\tilde{r}_{i\beta }/r_{i})^{2}$ are given by^46,61,62^: (0.4460,0.5571,0), (0.0992,0,0.9052), (0.7505,0.2461,0), (0.3976,0.2280,0.3722), and (0.7530,0,0.2538) corresponding to the hopping energy $t_{1}$, $t_{2}$, $t_{3}$, $t_{4}$, and $t_{5}$, respectively. So, it is clear how the strain comes into play role in deforming the hopping energies. On the other hand, following the Harrison rule $( t_{i}\propto r^{-2}_{i})$^63^, we obtain

11 $$\begin{aligned} \tilde{t}_{i}\approx \Big[ 1-2\sum _{\beta }\alpha _{i}^{\beta }\varepsilon _{\beta }\Big] t_{i}. \end{aligned}$$

**Figure 2:**
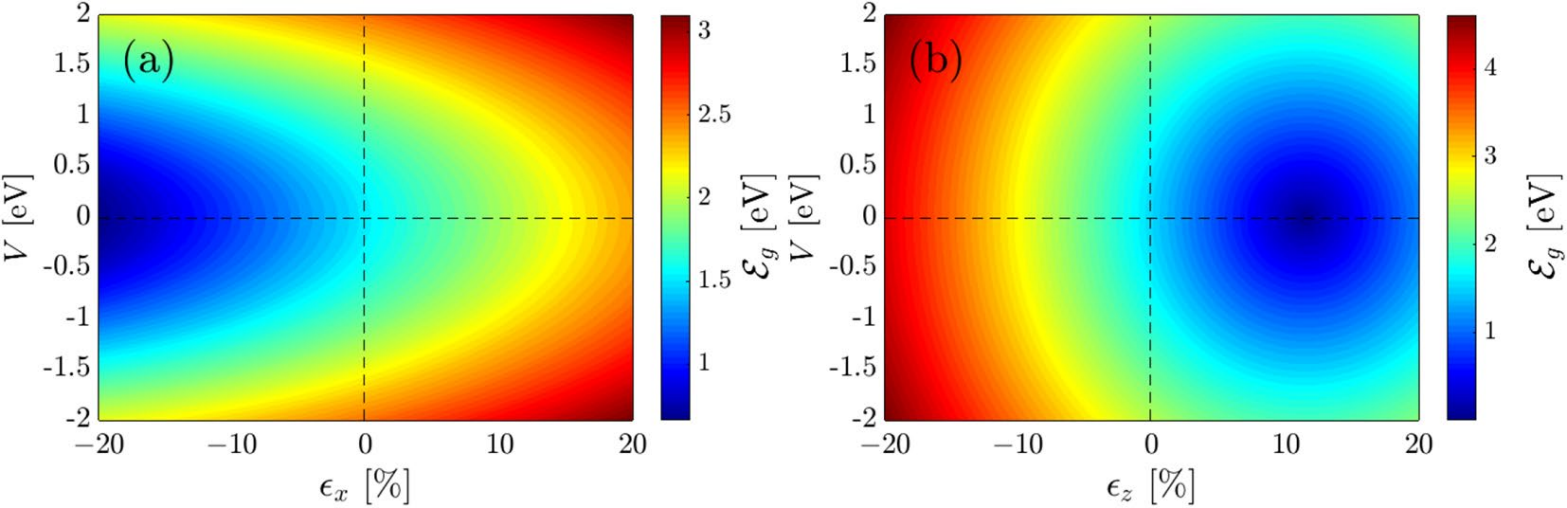
The variation of the band gap in the presence of gate voltages $-2$ eV $\leq V \leq+2$ eV associated with the compressive/tensile strain along the (**a**) *x*-direction and the (**b**) *z*-direction.

We first need to discuss the effect of both strain and gate on the electronic phase of phosphorene, since it will be connected directly to the optical properties. To do so, we focus on the band gap quantity as well as divide the following analysis into two parts, (1) in the presence of individual strain and gate voltage and (2) in the presence of both strain and gate simultaneously. The first part has been covered in the previous works^46,50,52^, while the second part has not reported/studied to date. It should be noted that the monolayer BP can not sustain the strains more than 30%^64^, for this reason, we work with the interval $[-20\%,+20\%]$ for both compressive and tensile strains along both in-plane and out-of-plane directions. Also, the gate potential is from $-2$ eV to $+2$ eV to be applied and accessible in the experiment.

In the presence of individual strains at $V=0$ eV, uniaxial ones are first discussed. In Fig. 2a, at $V=0$ eV and $\epsilon _x =0\%$, the band gap is 1.52 eV, while it linearly decreases [see Eq. (11)] with compressive $\epsilon _x <0$, while increasing linearly with tensile $\epsilon _x >0$. These, in turn, mean that a semiconductor-to-semimetal phase transition occurs for BP in the presence of compressive $\epsilon _x <0$, while phosphorene keeps its semiconducting phase with tensile $\epsilon _x >0$. We comment that the semimetal phase appears when the band gap becomes zero or negative. In Fig. 2b, at $V=0$ eV, the symmetry is broken down with $\epsilon _z$ such that the band gap increases with compressive $\epsilon _z < 0$, while decreases with $\epsilon _z > 0$ up to $\epsilon _z \simeq +12\%$ [at this critical strain, the band gap approaches zero], leading to a semiconductor-to-semimetal phase transition, and increases after that, meeting the initial semiconducting phase. In this case, the changes are linear as well. So, there is a critical point for out-of-plane strain at which the system suffers from a nontrivial phase transition. This strain modulus will also manifest itself in the optical properties.

Let us turn to the case of absence of strain $\epsilon _{x/y} =0\%$ and $\epsilon _z =0\%$ and presence of gate *V* only. In Fig. 2a, at $\epsilon _x =0\%$, the band gap symmetrically increases with both negative and positive gates, meaning that gate polarity and eventually reversing the current direction in the system does not affect the electronic phase of the system in the presented model, while it may affect if one considers the charge screening effect^58^ induced by gating. This similarly happens in Fig. 2b at $\epsilon _z =0\%$, i.e. the increasing rate of the band gap with $\pm V$ is the same, as expected.

**Figure 3:**
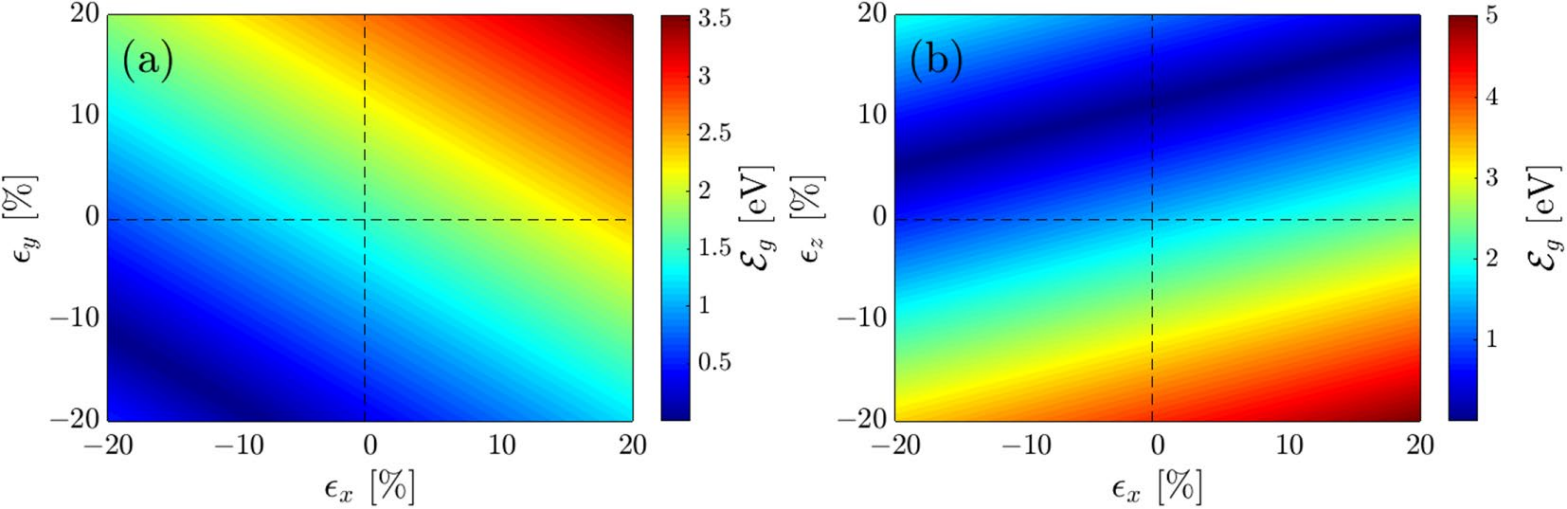
The band gap of monolayer phosphorene in the presence of both uni- and bi-axial compressive and tensile strains for (**a**) $\epsilon _y$-$\epsilon _x$ and (**b**) $\epsilon _z$-$\epsilon _x$.

Turn to the presence of the gate and strains simultaneously. Two types of strain and gate allow us to divide Fig. 2a into four parts, (1) $V >0$ and $\epsilon _x >0$, (2) $V >0$ and $\epsilon _x <0$, (3) $V <0$ and $\epsilon _x >0$, and (4) $V <0$ and $\epsilon _x <0$. Due to the symmetrical behavior of the band gap with gate sign, areas (1), and (4) as well as (2) and (3) behave similarly and for this reason, in the following, we only analyze one of the sets. We only analyze the diagonal part of these parts to avoid confusion. In the first/fourth area, the band gap increases, and a semiconductor-to-insulator phase transition is expected to appear at high-enough gate voltages as well as at strong-enough strains. In the second/third area, although the band gap increases again, its increasing rate is less than the first area, meaning that the gated system shows different responses to the compressive and tensile strains. The compressive strain tries to keep the semiconducting phase of the phosphorene, while the tensile strain changes the phase to the insulating one.

As for the presence of out-of-plane strain $\epsilon _z$, we also divide Fig. 2b into four parts, (1) $V>0$ and $\epsilon _z >0$, (2) $V>0$ and $\epsilon _z <0$, (3) $V<0$ and $\epsilon _z >0$, and (4) $V<0$ and $\epsilon _z <0$. Again, we only focus on (1) and (2) here. For the first/fourth area, one observes that the band gap does not change significantly and decreases (increases) before (after) the critical strain $\epsilon _z \simeq +12\%$, as expected, and the system remains in its initial phase anyway. Whereas it increases in the second/third area, resulting in a semiconductor-to-insulator phase transition. These competitions are important in the optoelectronics in which the interband optical transitions strongly depend on the band gap changes.

Another possible perturbation is biaxial strain in the absence and presence of gate voltage. Since the band gap changes linearly with the gate voltage, we would focus on the absence of a gate when applying biaxial strains in Fig. 3. Here, we only need to analyze the uniaxial strain $\epsilon _y$ in Fig. 3a. At $\epsilon _x = 0\%$, the band gap linearly increases and decreases, respectively, with tensile $\epsilon _y > 0$ and compressive $\epsilon _y < 0$ similar to the case of $\epsilon _x$, but with a bit larger modules. Next, we divide Fig. 3a into four areas, (1) $\epsilon _x > 0$ and $\epsilon _y > 0$, (2) $\epsilon _x < 0$ and $\epsilon _y > 0$, (3) $\epsilon _x < 0$ and $\epsilon _y < 0$, and (4) $\epsilon _x > 0$ and $\epsilon _y < 0$. For biaxial effects, we only walk on the diagonal blocks. In the first region, the band gap increases if the in-plane strain is tensile for both components, leading to a semiconductor-to-insulator phase transition. On the other hand, it decreases in the third region when both components of the in-plane strain are compressive, leading to a semiconductor-to-semimetal phase transition at $\epsilon _x \simeq -12\%$ and $\epsilon _y \simeq -8\%$. After these critical in-plane strains, the band gap starts to increase slightly, keeping the semiconducting phase of the system. Simply, one observes that for the second and fourth cases, the band gap is not changed significantly and phosphorene is still a semiconductor.

As soon as the out-of-plane strain is switched on, i.e. $\epsilon _z$, a strong competition starts with the in-plane ones in tuning the band gap of puckered BP. In Fig. 3b, the biaxial strains are again along the diagonal line of blocks. In the first block, $\epsilon _x > 0$ and $\epsilon _z > 0$, the band gap decreases slightly and the system transits to the semimetallic phase, while it increases slightly in the fourth block, $\epsilon _x > 0$ and $\epsilon _z < 0$, and the insulating phase comes up. In the second block, $\epsilon _x < 0$ and $\epsilon _z > 0$, the band gap decreases and increases before and after the minimum band gap and a semiconductor-to-semimetal-to-semiconductor phase transition emerges, whereas in the third block, $\epsilon _x < 0$ and $\epsilon _z < 0$, the band gap increases slightly and the semiconducting phase is preserved.

## Interband optical conductivity, dielectric function and EELS of phosphorene

Although in the presence of strain, one would neglect the intraband optical transitions, it might not be the case for the applied gate voltages. As mentioned before, for the charge screening effects by gating, one should include the intraband transitions in the theory^58^, however; such an effect does not matter in our applied gate. From these points, in the following, we only focus on the interband optical transitions. In this section, we intend to turn to the live subject of the manuscript using the Kubo formula^65–67^ to calculate the optical conductivity of phosphorene including both intraband and interband excitations when it is subjected to an applied optical field $\mathbf {E}(\omega ,t)$ with frequency $\omega$.

The Hamiltonian introduced in Eq. (8) is so-called the non-interacting Hamiltonian in the absence of the optical field (OF), however, considering the external field $\mathbf {E}(\omega ,t)$ as the perturbation, the perturbed/interacting Hamiltonian, describing the interaction between the optical field and fermions in phosphorene is given by

12 $$\begin{aligned} \mathcal {H}=\mathcal {H}(\mathbf {k})+\mathcal {H}_{\text {OF}}, \end{aligned}$$

where $\mathcal {H}_{\text {OF}}=\mathbf {J}\cdot \mathbf {A}$ is the perturbation Hamiltonian including the vector potential $\mathbf {A} = - \int \mathbf {E}(\omega ,t) dt$ and the current density of host fermions $\mathbf {J} = \sigma \mathbf {E}(\omega ,t)$ is related to the optical field using the optical conductivity tensor $\sigma$. Consisting of the converted wave-vectors $\mathbf {k}+(e/\hbar )\mathbf {A}$, the $\alpha$-component of $\mathbf {J}$ is obtained

13 $$\begin{aligned} J_{\alpha }=-\frac{e}{\hbar }\sum _{\mathbf {k},s}\hat{f}^{\dagger }_{\mathbf {k},s}\hat{f}_{\mathbf {k},s}\mathcal {J}^{\alpha }_{\mathbf {k}}+\texttt {i}\frac{e}{\hbar }\sum _{\mathbf {k},s}\hat{f}^{\dagger }_{\mathbf {k},s}\hat{f}_{\mathbf {k},-s}\mathcal {K}^{\alpha }_{\mathbf {k}}, \end{aligned}$$

where $\{\mathcal {J}^{\alpha }_{\mathbf {k}},\mathcal {K}^{\alpha }_{\mathbf {k}}\}$ are the velocity of fermions in phosphorene subjected to the optical field. We obtain^19,46,56,57^

14a $$\begin{aligned} \mathcal {J}^{x}_{\mathbf {k}}&=+2\tilde{t}_{1}a_{1x}\sin \left( k_{x}a_{1x}+\theta _{\mathbf {k}}\right) +\tilde{t}_{2}a_{2x}\sin \left( k_{x}a_{2x}-\theta _{\mathbf {k}}\right) +2\tilde{t}_{3}a_{3x}\cos \left( k_{y}b/2\right) \sin \left( k_{x}a_{3x}-\theta _{\mathbf {k}}\right) \nonumber \\&\quad +2\tilde{t}_{4}a\sin \left( k_{x}a/2\right) \cos \left( k_{y}b/2\right) +\tilde{t}_{5}a_{5x}\sin \left( k_{x}a_{5x}+\theta _{\mathbf {k}}\right) , \end{aligned}$$

14b $$\begin{aligned} \mathcal {J}^{y}_{\mathbf {k}}&=+b\tilde{t}_{1}\sin \left( k_{y}b/2\right) \cos \left( k_{x}a_{1x}+\theta _{\mathbf {k}}\right) +b\tilde{t}_{3}\sin \left( k_{y}b/2\right) \cos \left( k_{x}a_{3x}+\theta _{\mathbf {k}}\right) +2\tilde{t}_{4}b\cos \left( k_{x}a/2\right) \sin \left( k_{y}b/2\right) , \end{aligned}$$

14c $$\begin{aligned} \mathcal {K}^{x}_{\mathbf {k}}&=-2\tilde{t}_{1}a_{1x}\cos \left( k_{y}b/2\right) \cos \left( k_{x}a_{1x}+\theta _{\mathbf {k}}\right) +\tilde{t}_{2}a_{2x}\cos \left( k_{x}a_{2x}-\theta _{\mathbf {k}}\right) +2\tilde{t}_{3}a_{3x}\cos \left( k_{y}b/2\right) \cos \left( k_{x}a_{3x}-\theta _{\mathbf {k}}\right) \nonumber \\&\quad -\tilde{t}_{5}a_{5x}\cos \left( k_{x}a_{5x}+\theta _{\mathbf {k}}\right) , \end{aligned}$$

14d $$\begin{aligned} \mathcal {K}^{y}_{\mathbf {k}}&=+b \tilde{t}_{1}\sin \left( k_{y}b/2\right) \sin \left( k_{x}a_{1x}+\theta _{\mathbf {k}}\right) -b \tilde{t}_{3}\sin \left( k_{y}b/2\right) \sin \left( k_{x}a_{3x}-\theta _{\mathbf {k}}\right)\, , \end{aligned}$$

where $a_{1x} \simeq 1.41$ Å  and $a_{2x}\simeq 0.79$ Å, $a_{3x}=a_{1x}+2a_{2x}$, $a_{4x}=a_{1x}+a_{2x}$ , and $a_{5x}=2a_{1x}+a_{2x}$. Also, $\theta _{\mathbf {k}} = -\mathtt{i} \log (\sqrt{g_{\mathbf {k}}/g^*_{\mathbf {k}}})$. These are the expressions in the absence of electric field, while including *V* makes it complicated. For this reason, we have numerically calculated these velocities.

After substituting Eqs. (14a–14d) into Eq. (13) and then using the linear response theory for the optical conductivity

15 $$\begin{aligned} \sigma _{\alpha \beta }(\omega )=\frac{1}{\hbar \omega }\int ^{\infty }_{0}dt e^{\texttt {i}\omega t}\left\langle \left[ J_{\alpha }(t),J_{\beta }(0)\right] \right\rangle , \end{aligned}$$

we obtain the total optical conductivity. Thus, the interband optical transitions along the $\alpha$ direction can be calculated through

16 $$\begin{aligned} \frac{\sigma ^{\texttt {inter}}_{\alpha \alpha }(\omega )}{\sigma _{0}}={}\frac{2 \mathtt{i}}{\hbar \omega } \sum _{\mathbf {k}\in \text {FBZ}} (\mathcal {K}^{\alpha }_{\mathbf {k}})^2 \left[ \frac{n^{\text {FD}}_{\mathbf {k},+}-n^{\text {FD}}_{\mathbf {k},-}}{\hbar \omega +\left( \mathcal {E}_{+}(\mathbf {k})-\mathcal {E}_{-}(\mathbf {k})\right) +\mathtt{i}\eta }-\frac{n^{\text {FD}}_{\mathbf {k},+}-n^{\text {FD}}_{\mathbf {k},-}}{\hbar \omega -\left( \mathcal {E}_{+}(\mathbf {k})-\mathcal {E}_{-}(\mathbf {k})\right) +\mathtt{i}\eta }\right] , \end{aligned}$$

where $\sigma _{0}=e^{2}/\hbar$ is the universal value for the optical conductivity and $\eta =10$ meV refers to the finite damping between the valence and conduction bands. Furthermore, $n^{\text {FD}}_{\mathbf {k},\pm }=1/1+exp\left[ ({\mathcal {E}}_{\pm }(\mathbf {k})-\mu )/k_{\text {B}}T\right]$ is the Fermi-Dirac distribution function at the chemical potential $\mu$ [$k_{\text {B}}$ being the Boltzmann constant] and temperature *T*. It should be pointed out that the Hall conductivities are zero, i.e. $\sigma _{xy}(\omega )=\sigma _{yx}(\omega )=0$ because of the puckered structure symmetry of phosphorene.

For the present paper, we do not focus on the interband optical conductivity itself, since it has been well-addressed in the previous works^46,47,50–52^. The investigation of the EELS spectra in gated and strained BP is the main aim of the present work and, in doing so, one needs the dielectric function, given by

17 $$\begin{aligned} \varepsilon ^{\texttt {inter}}_{\alpha \alpha }(\omega )=\varepsilon _r+\frac{\mathtt{i}\sigma ^{\texttt {inter}}_{\alpha \alpha }(\omega )}{\omega \varepsilon _0 d_{BP}}, \end{aligned}$$

where the thickness of monolayer BP is $d_{BP}\simeq 0.7$ nm^39^. On the other hand, $\varepsilon _r=5.65 (\varepsilon _0)$ is the relative (vacuum) permittivity of single-layer BP^25^. Finally, the EELS can be calculated as

18 $$\begin{aligned} L^{\texttt {inter}}_{\alpha \alpha }(\omega )=-\mathrm{Im}\left[ \frac{1}{\varepsilon ^{\texttt {inter}}_{\alpha \alpha }(\omega )}\right] . \end{aligned}$$

**Figure 4:**
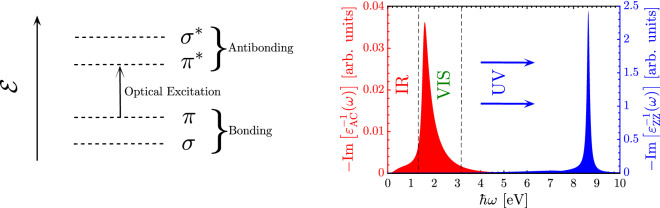
Left side, general interband optical transitions and excitations between different bonding and antibonding electronic orbitals in phosphorene. Right side, electron energy loss spectrum of “pristine” monolayer BP along both AC and ZZ directions inside the infrared (IR), visible (VIS), and ultraviolet (UV) regions of the electromagnetic spectrum.

Thus, it is straight forward to calculate EELS of phosphorene along both armchair (AC) and zigzag (ZZ) directions having interband optical conductivity. The interband transitions and excitations in our 2D condensed matter system depend strongly on the energy level stemming from the highest valence bands and the lowest conduction bands, as shown in the left panel of Fig. 4.

As mentioned before, EELS provides useful information on how the energy of scattered host electrons is changed from the incident light and external perturbations. Also, from an experimental point of view, EELS directly probes the loss function of a material^55^ to understand the single-particle, excitons, and plasmons. It is worth noting that the EELS behavior of phosphorene originates strongly from the energy dispersion of phosphorene, for this reason, one expects different responses in EELS along different directions, as shown in the right panel of Fig. 4, in good agreement with Refs.^68,69^. In other words, the intrinsic anisotropic feature in phosphorene is manifested in the EELS diagram. As can be seen, EELS of phosphorene along the AC direction shows its maximum intensity corresponding to the maximum response to frequencies inside the visible (VIS) region of the electromagnetic spectrum, while this happens inside the ultraviolet (UV) region along the ZZ direction. Also, small intensities can be observed inside the infrared (IR) region along the AC direction. This, in turn, confirms highly anisotropy optical excitations in phosphorene. It should be pointed out that the incident momentum of optical light is not considered and approximated as $\mathbf {q} \rightarrow 0$ since the maximum intensity among all the momentum-dependent EELS peaks is related to this limit of $\mathbf {q}$^68^, which its non-zero value is interesting for plasmonics.

## Results and discussions

Here we apply the formalism introduced previously to possible configurations of the presence of strain and electric field. Importantly, we would stress that, although our tight-binding method is very insightful, it may fail in detecting the observed experimentally low (high)-energy intraband (interband) plasmons involving transitions across various energy bands. This originates from the two-band model used here, however, it would work out well for the low-energy interband excitations. Indeed, the low-energy intraband plasmon spectrum has been investigated in Refs.^70,71^. On the other hand, the high-energy interband transitions correspond to the ZZ direction, while the low-energy ones belong to the AC direction. These all are in an excellent agreement with recent measurements^72–74^.

**Figure 5:**
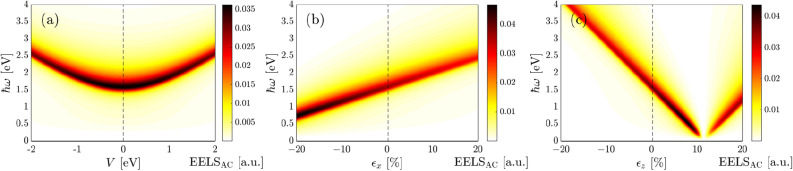
EELS (in arbitrary units) of monolayer perturbed BP along the AC direction as functions of the incident optical energy $\hbar \omega$, (**a**) gate voltage *V* in the absence of strain $\epsilon _x = \epsilon _y = \epsilon _z = 0$, (**b**) strain $\epsilon _x$ at $V=0$ eV and $\epsilon _y = \epsilon _z = 0$ and (**c**) strain $\epsilon _z$ at $V=0$ eV and $\epsilon _x = \epsilon _y = 0$.

**Figure 6:**
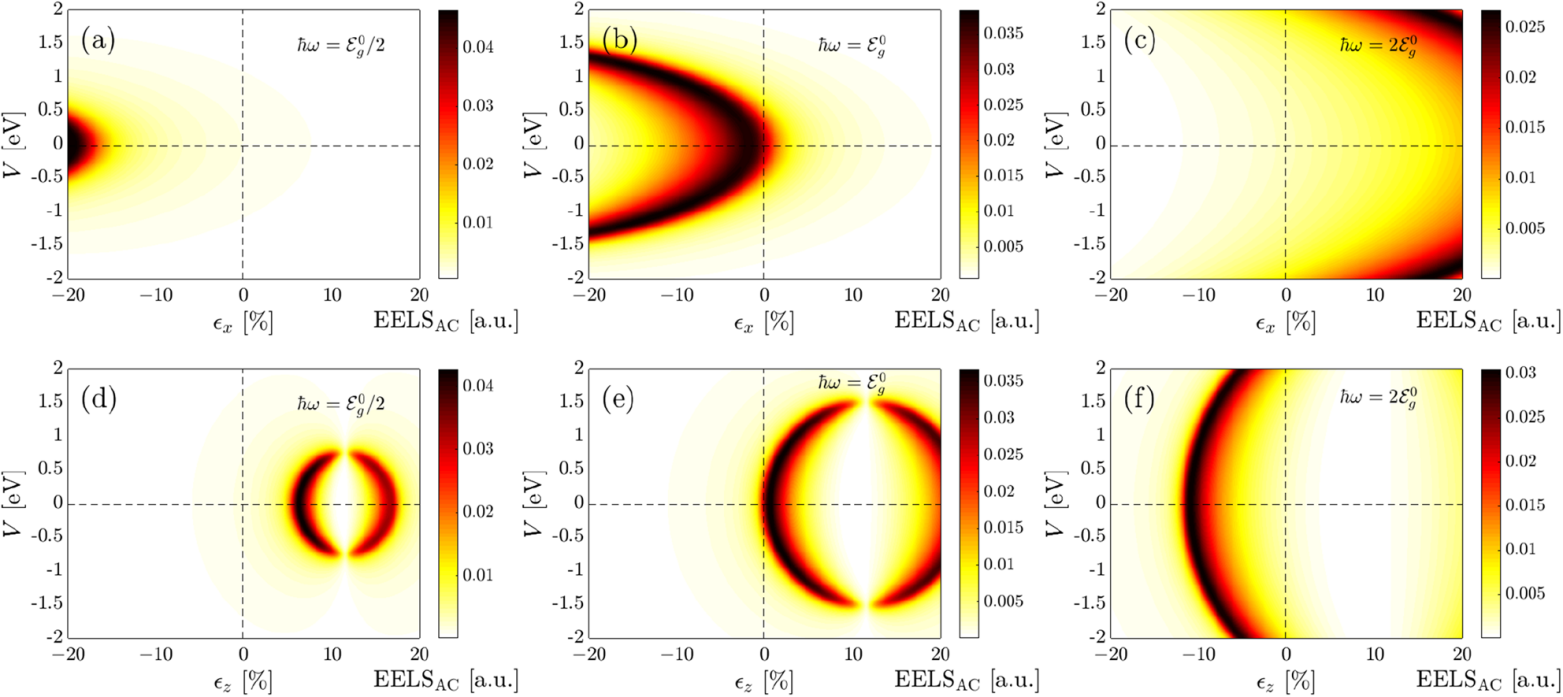
Low-energy interband part of the EELS in phosphorene along the AC direction for the combined effects of *V* and $\epsilon _x$ at the optical energy (**a**) $\hbar \omega = \mathcal {E}^0_g/2$, (**b**) $\hbar \omega = \mathcal {E}^0_g$ and (**c**) $\hbar \omega = 2\mathcal {E}^0_g$. Corresponding results for the combined effects of *V* and $\epsilon _z$ are presented in (**d**–**f**).

**Figure 7:**
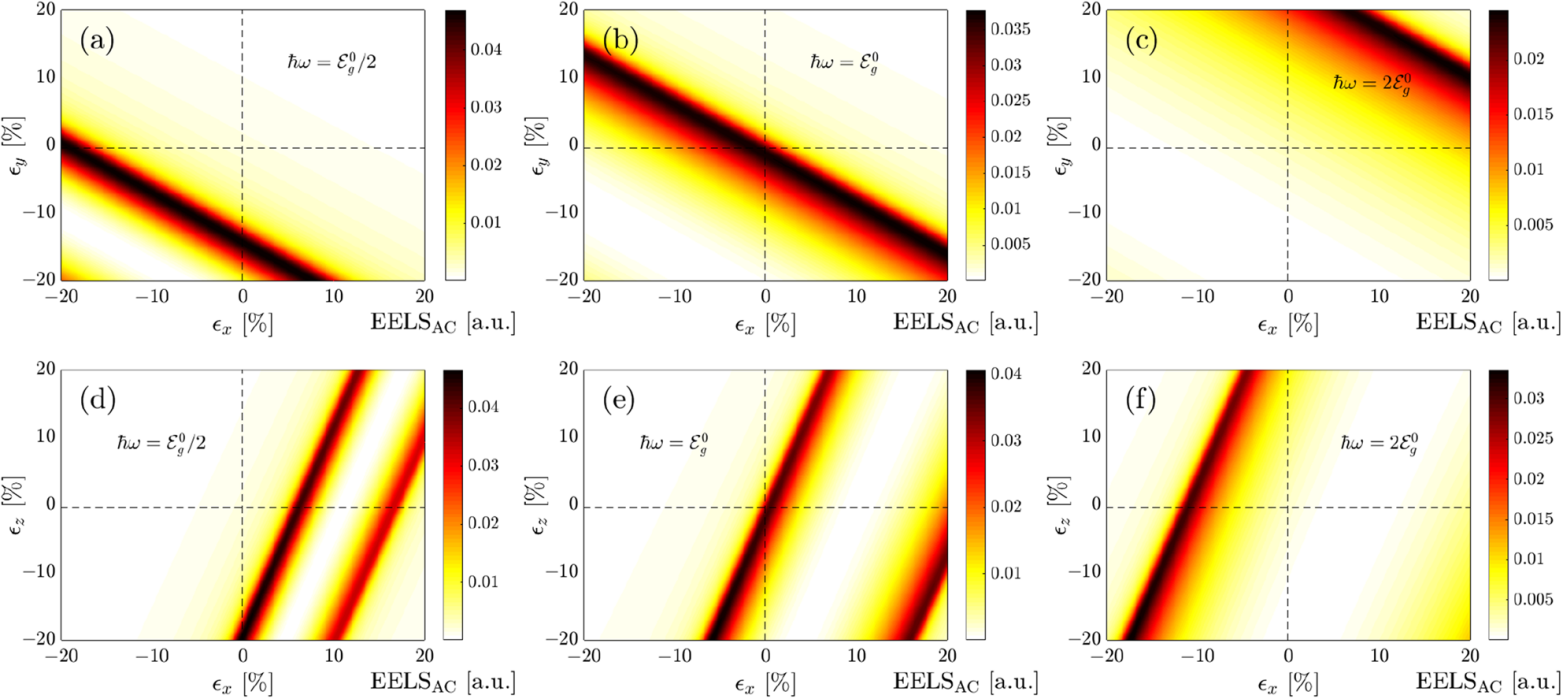
Low-energy interband part of the EELS in phosphorene along the AC direction for the combined effects of $\epsilon _y$ and $\epsilon _x$ at the optical energy (**a**) $\hbar \omega = \mathcal {E}^0_g/2$, (**b**) $\hbar \omega = \mathcal {E}^0_g$ and (**c**) $\hbar \omega = 2\mathcal {E}^0_g$. Corresponding results for the combined effects of $\epsilon _z$ and $\epsilon _x$ are presented in (**d**–**f**).

We remind that in addition to the optical energy $\hbar \omega$, the strain modulus and gate voltage are also present in the system and the response of the scattered host electrons from the incident light depends strongly on these parameters as well. First of all, the competition between the optical energy and strain/gate voltage is discussed in Fig. 5. Then, individual and combined gate voltage and uniaxial strain effects on EELS for $\hbar \omega < \mathcal {E}_g$, $\hbar \omega = \mathcal {E}_g$ and $\hbar \omega > \mathcal {E}_g$ are addressed in Fig. 6. Finally, in Fig. 7, biaxial strain effects in the absence of gate voltage for different possible optical energies above-mentioned will fully be explained. These altogether refer to the systematical findings, which have not yet been reported theoretically. It is necessary to mention that the temperature is set to 10 K in the present work. Also, we comment that the system is undoped at all and there is no temperature-dependent Fermi energy and doping effect. This, in turn, means that the optical excitation does not depend on the temperature and doping concentration in this formalism.

Having discussed the band gap variations in Figs. 2 and 3 with strain and gate voltage based on the low-energy band structure in strained and gated single-layer BP, as well as, after excellently reproducing low-energy interband excitations, we turn to the corresponding specific features of the perturbed EELS in phosphorene when the optical energy $\hbar \omega$ intends to alter the basic perturbed electro-optical properties. Note that although the optical energy varies from 0 to 4 eV to cover the IR and VIS alterations of EELS, the results up to $\hbar \omega \simeq 3.2$ eV relate to the low-energy transitions. From Eq. (16), the interband optical conductivity clearly proportions to energy difference $\mathcal {E}_{+}(\mathbf {k})-\mathcal {E}_{-}(\mathbf {k})$. However, it should be pointed out that this statement is valid for finite energy difference making the interband excitations meaningful.

As shown in Fig. 5a, the maximum EELS appears for energies near the band gap of the pristine BP, i.e. when $V=0$ eV. As expected, it decreases with optical energy when getting away from the VIS region. However, turning on the gate voltage, independent of the polarity, and in other words independent of the carrier current direction, the intensity of interband excitations decreases slightly symmetrically considering the zero gate voltage and the EELS peak position shifts to the higher optical energies. This, in turn, means that the EELS is blue-shifted and slightly its strength is lost^68^. We comment that energies below the pristine band gap (1.52 eV) correspond to the intraband excitations, which is not considered in our formulation [see the previous reasons] and one may observe them in DFT or experimental works. For energies greater than twice the band gap, there is almost no intensities for EELS.

Interestingly, a different type of shift is observed as the uniaxial strain $\epsilon _x$ is applied. As represented in Fig. 5b, the maximum EELS intensity at an energy equal to the pristine band gap increases further with compressive strain, while it decreases with tensile strain, accompanied by a blue shift (towards the higher energies) for EELS with $\epsilon _x>0$ and a red shift (towards the lower energies) with $\epsilon _x<0$. In this case, the EELS variation is not symmetric regarding the type of strain, compressive or tensile, in contrast to the gate voltage. These can be understood from the band gap variations since the interband optical transitions depend strongly on the difference between the lowest conduction band and the highest valence band (see the left panel of Fig. 4). These results report that the intraband excitations may be affected significantly by the uniaxial strain along the *x*-direction.

Turning to the out-of-plane strain effects on the EELS of phosphorene in Fig. 5c. We remember that the band gap found its minimum value at the critical strain $\epsilon _z \simeq +12$ %. Thus, one expects no interband transitions at this strain. As mentioned before, this stems from the basic physics of EELS and optical excitations implemented in the Kubo formula. At this strain, valence and conduction bands almost touch each other and there is no interband transitions, from this point, no EELS response comes up. As can be seen, this is the case independent of the optical energy and interesting behaviors are observed for EELS in the presence of compressive and tensile strains, in contrast to the in-plane strains. Although the EELS intensity decreases with compressive $\epsilon _z <0$, it is blue-shifted and one may propose the out-of-plane strain to tune the high-energy interband transitions. The most interesting behavior relates to the tensile out-of-plane strain. It is noticeable that the EELS intensity increases up to the above-mentioned critical $\epsilon _z \simeq +12$ %, it disappears and reappears after this strain, resulting in an EELS intensity increase gradually. So, a red shift and blue shift for EELS of BP in the presence of tensile $\epsilon _z$ is the direct consequence of these achievements.

So far we focused on the individual contributions of gate voltage and uniaxial strain. Having established these foundations, let us turn to the combined effects of gate voltage and uniaxial in- and out-of-plane strains in Fig. 6. Similar to the band gap analysis, we divide each panel into four regions, (1) $V >0$ and $\epsilon _{x/z} >0$, (2) $V >0$ and $\epsilon _{x/z} <0$, (3) $V <0$ and $\epsilon _{x/z} >0$, and (4) $V <0$ and $\epsilon _{x/z} <0$. In contrast to the previous figure, we have to focus on special optical energies to find the competition between gate and strains in tuning the EELS of BP. To this end, we should not exceed the critical optical energy of the VIS region, i.e. $\simeq$ 3.2 eV. For this reason, three different frequencies $\hbar \omega = \mathcal {E}^0_g/2$, $\hbar \omega = \mathcal {E}^0_g$ and $\hbar \omega = 2\mathcal {E}^0_g$ are examined in the following corresponding to 0.76 eV, 1.52 eV and 3.04 eV, respectively. Although some information may be re-found in the previous plot, the combined effects are necessary to be addressed as the main novelty of the present paper.

At first glance, one would notice that at $\hbar \omega = 0.76$ eV, among gate voltage and strain, a strain involves the EELS response in BP. In turn, among all possible strains, the compressive in-plane and tensile out-of-plane strains contribute only (see Fig. 5b,c). However, the situation may be different if both gate and strain are present as external perturbations, as illustrated in Fig. 6a,d. First of all, one observes that the EELS response is only highlighted the compressive in-plane and tensile out-of-plane strains. Second, maximum EELS emerges at strong-enough compressive in-plane strains and gate voltage leads to the EELS reduction in this region, meaning that the intensity of the red shift decreases, see Fig. 6a. Note that a small (large) band gap corresponds to the strong (weak) EELS response. This can be understood from this fact that the distance between the lowest conduction band and the highest valence band increases with gate voltage, leading to the reduction of EELS response, because of the untouched bands in contrast to the case of strain $\epsilon _z$. As for the EELS behaviors in Fig. 6d, we conclude that zero EELS at $\epsilon _z \simeq +12\%$ never becomes finite, however; EELS before this critical strain is greater than EELS after that. On the other hand, both sides decrease with the gate voltage, as expected, supporting a narrow range of tensile out-of-plane strain. Generally, at a fixed in-plane (out-of-plane) strain $\epsilon _x<-18\%$ ($+5\%<\epsilon _z<+18\%$), EELS intensity increases and decreases anisotropically.

In the resonance case, $\hbar \omega = 1.52$ eV, where the optical energy coincides approximately with the energy gap of pristine BP, we identify the competition between gate voltage and strains in Fig. 6b,e. As shown in Fig. 6b, second and third regions are most affected, while the first and fourth ones are affected rarely, as expected. At this energy, although the EELS intensity is reduced slightly with $\pm V$ and $\epsilon _x <0$, a smoother decreasing trend for the EELS intensity appears for $\epsilon _x >0$ as well. Thus, although the EELS response is symmetric concerning the gate voltage, this is not the case for strain along the *x*-direction. On the diagonal part of the second and third regions, however, the EELS intensity remains almost constant up to a critical voltage of $<1.5$ eV and decreases immediately. Similarly, at a fixed strain $\epsilon _x<0\%$, the EELS intensity increase and decreases from $V<0$ to $V>0$. Highly anisotropic EELS responses to the in-plane and out-of-plane strains appear in Fig. 6e for which the zero EELS intensity around the critical strain $\epsilon _z \simeq +12\%$ is wider than frequencies below the pristine gap, meaning that system responds to the light more in this energy covering a wider range of gate voltage. This can be traced back to the band gap changes provided in Fig. 2a, as a different range of allowed gate voltages appears depending on the strain modulus. For $\epsilon _z<0$ there is almost no EELS response. At fixed strains $0<\epsilon _z<+10\%$ and $\epsilon _z>+12\%$, EELS intensity increases and decreases from $V<0$ to $V>0$ symmetrically.

Moving towards the higher optical energies $\hbar \omega = 3.04$ eV, the prominent EELS responses are limited. The corners of the first and fourth regions in Fig. 2a as well as the second and third regions of Fig. 6f show maximum responses symmetrically. These are in contrast to the low optical energies (see Fig. 6a,d). The EELS responses at second and third [first and third] regions of Fig. 6c [(f)] are almost similar and no highlight is needed to be mentioned there. At fixed strain within the interval $0<\epsilon _x<+10\%$, EELS response decreases (increases) slightly with gate voltage $V<0$ ($V>0$), while for $\epsilon _x>+10\%$, the decreasing and increasing rates are stronger (see Fig. 2a). Comparing the EELS response at $+V$ and $-V$ at a fixed out-of-plane strain in the range of $-13\%<\epsilon _z<0$, depending on the gate voltage, intensity decreases and increases with $V<0$ and $V>0$, respectively, as expected. Also, on the diagonal lines of these regions, an increasing trend is observed as both gate voltage and strains are increased simultaneously.

Finally, we calculate the EELS function when the biaxial strains are present. In Fig. 7 we plot EELS response for two biaxial strains $\epsilon _y - \epsilon _x$ and $\epsilon _z - \epsilon _x$ in the presence of three different optical frequencies. Calculating EELS response is a straightforward process as the previous cases, however, the results are different and need to be discussed.

A plot in Fig. 7a–c is useful to see with clarity which in-plane component of applied strain is likely to give the maximum EELS response. Frequencies below the pristine band gap produce the largest contribution to the EELS intensity in the third region where both in-plane components are compressive, while rarely (almost zero) contribution is observed in the first region. However, for very small tensile strain $\epsilon _y$ and small/intermediate strain $\epsilon _x$, EELS intensity is maximum as well. Interestingly, the EELS variation with in-plane strains is linear, as expected from previously linear behavior of the strained band gap. The dominant contribution of in-plane strains in EELS response for the optical energy $\hbar \omega \simeq \mathcal {E}^0_g$ is obtained from the second and fourth regions. Also, small compressive strains contribute to EELS maximum at this optical energy. Notably, we see that the EELS response intensity for optical energies above the band gap is primarily dominated by the first region corresponding to the tensile in-plane strains. This was expected since at $\hbar \omega > \mathcal {E}^0_g$, the required bands for the interband transitions, and eventually the optical excitations are limited. In this regard, the new semimetallic phase of the system is attributed to the maximum EELS. Critical strains in all three panels for which the largest EELS intensity is reached can be observed in plots. Note that the underlying physical mechanism behind the EELS intensities is ascribed to the breaking of the symmetry between bonding energies between nearest atoms through a reconstruction of the intrasublayer and intersublayer hopping energies.

It is necessary to mention that, although the compressive strains $\epsilon _x$ were the only possible cases in EELS response at $\hbar \omega \simeq 0.76$ eV, the tensile ones come also into play role when $\epsilon _y$ is present as well. On the other hand, at $\hbar \omega \simeq 1.52$ eV, the role of tensile $\epsilon _x$ was not significant, while its contribution is dominant compared to the compressive $\epsilon _x$ as $\epsilon _y$ is switched on. However, at $\hbar \omega \simeq 3.04$ eV, not a significant insight is seen in the presence of $\epsilon _y$.

As for the out-of-plane strain contribution to the EELS intensity, Fig. 7d–f are plotted to present the role of vertical bond length change of atoms for different optical energies. Interestingly, although no EELS response was observed before when the out-of-plane strains were applied solely, this may not be the case in the presence of in-plane $\epsilon _x$, as can be seen in these panels. This means that the Kubo formula revalidates if $\epsilon _x$ is accompanied by $\epsilon _z$, resulting in deviations from the expectations. At $\hbar \omega \simeq 0.76$ eV, from Fig. 5c, one finds that the most contribution of EELS response is referred to the tensile $\epsilon _z$, while Fig. 7d reports a significant role of compressive $\epsilon _z$ as the $\epsilon _x$ is switched on. On the other hand, at $\hbar \omega \simeq 1.52$ eV, the role of tensile $\epsilon _z$ is a bit larger than compressive $\epsilon _z$, whereas Fig. 7e also shows a significant contribution of compressive $\epsilon _z$ as soon as the $\epsilon _x$ is coming up. At $\hbar \omega \simeq 3.04$ eV, the compressive $\epsilon _z$ were controlling the EELS response, while the tensile one becomes important as well when both in- and out-of-plane strains are present (see Fig. 7f).

In Fig. 7d, we present our calculations of EELS intensity for combined effects of in-plane and out-of-plane strains at $\hbar \omega = 0.76$ eV. The findings show that the first and fourth regions contribute mostly to the optical excitations and at several in- and out-of-plane strains, the band gap vanishes, and EELS approaches zero inside an oblique ribbon. This ribbon becomes more widespread as the optical energy increases (see Fig. 7e,f) since the intensity goes above the maximum peak and the bands at large enough energies are extremely limited. While in Fig. 7e, first, third and fourth regions are involved in the optical excitations, in Fig. 7f, most contributions of strains are dedicated to the second and third regions. Thus, EELS intensity is easily controlled depending on the competition between strains.

## Conclusions

In summary, we have employed the Kubo formula accompanied by the two-band tight-binding approach to study the strain and electric field stimuli on the EELS response of monolayer phosphorene. Particularly, we have studied the EELS response of phosphorene to an incident optical light in three different scenarios: (1) the individual effect of in- and out-of-plane strains, (2) the individual effect of the electric field, and (3) the combined effect of strain and electric field.

Through simple analysis, here we have showed unambiguously that the individual electric field leads to the band gap increasing, whereas the uniaxial compressive (tensile) in-plane strain displays a decreasing (increasing) trend for the band gap. The out-of-plane strains, however, lead to the appearance of different gaps and a critical strain $+12\%$ under which the semiconducting phase of monolayer BP transits to the semimetallic phase. A more complicated electronic phase in perturbed phosphorene with both electric field and strain simultaneously is the direct consequence of perturbed orbital hybridization.

The EELS response showed that the infrared and visible (ultraviolet) region is dedicated to the armchair (zigzag) direction; an intrinsic property of monolayer phosphorene. The results showed that the EELS of monolayer BP for both in-plane and out-of-plane strains may show broad [both low and high intra- and inter-band] excitonic and plasmonic structure which may be attributed to the collective excitations of both $\sigma$ and $\pi$ electrons, while the electric field is only attributed to the low-energy interband ones. Our results are consistent with recent reports on the optical properties of phosphorene. We have proposed different scaling procedures for strain and the electric field that allow us to effectively reach larger or smaller EELS responses inside the infrared and visible regions. Although in the case of individual stimuli, we have found a blue shift for electric field-induced phosphorene, a compressive/tensile (tensile/compressive) in-plane/out-of-plane strain leads to a red (blue) shift in the interband optical excitations. On the other hand, these shifts turn out to be controlled by the combined effects of stimuli. Consistent arguments based on the interband transitions indicate that for the critical out-of-plane strain $\epsilon _z\simeq +12\%$ the Kubo theory breaks down in the presence of semiconductor-to-metal phase transition for which EELS response vanishes. Our results pave the way for the applications including simultaneous determination of EELS response in electric field-induced and strained single-layer BP.

## Data Availability

The data that support the findings of this study are available from the corresponding author upon reasonable request.
